# Identification of Two Kinase Inhibitors with Synergistic Toxicity with Low-Dose Hydrogen Peroxide in Colorectal Cancer Cells In vitro

**DOI:** 10.3390/cancers12010122

**Published:** 2020-01-02

**Authors:** Eric Freund, Kim-Rouven Liedtke, Lea Miebach, Kristian Wende, Amanda Heidecke, Nagendra Kumar Kaushik, Eun Ha Choi, Lars-Ivo Partecke, Sander Bekeschus

**Affiliations:** 1 Department of General, Visceral, Thoracic and Vascular Surgery, Greifswald University Medical Centre, 17475 Greifswald, Germany; eric.freund@inp-greifswald.de (E.F.); Kim.Liedtke@med.uni-greifswald.de (K.-R.L.); miebachlea.uni@gmail.com (L.M.); partecke@googlemail.com (L.-I.P.); 2 Centre for Innovation Competence (ZIK) *Plasmatis*, Leibniz Institute for Plasma Science and Technology (INP Greifswald), 17489 Greifswald, Germany; Kristian.wende@inp-greifswald.de (K.W.); amanda.k.heidecke@gmail.com (A.H.); 3 Plasma Bioscience Research Center (PBRC), Kwangwoon University, Seoul 139-710, Korea; kaushik.nagendra@kw.ac.kr (N.K.K.); choipdp@gmail.com (E.H.C.); 4 General-, Visceral-, and Thoracic Surgery, Helios Clinic Schleswig, 24837 Schleswig, Germany

**Keywords:** anticancer drugs, pancreatic cancer, screening, tumor spheroids

## Abstract

Colorectal carcinoma is among the most common types of cancers. With this disease, diffuse scattering in the abdominal area (peritoneal carcinosis) often occurs before diagnosis, making surgical removal of the entire malignant tissue impossible due to a large number of tumor nodules. Previous treatment options include radiation and its combination with intraperitoneal heat-induced chemotherapy (HIPEC). Both options have strong side effects and are often poor in therapeutic efficacy. Tumor cells often grow and proliferate dysregulated, with enzymes of the protein kinase family often playing a crucial role. The present study investigated whether a combination of protein kinase inhibitors and low-dose induction of oxidative stress (using hydrogen peroxide, H_2_O_2_) has an additive cytotoxic effect on murine, colorectal tumor cells (CT26). Protein kinase inhibitors from a library of 80 substances were used to investigate colorectal cancer cells for their activity, morphology, and immunogenicity (immunogenic cancer cell death, ICD) upon mono or combination. Toxic compounds identified in 2D cultures were confirmed in 3D cultures, and additive cytotoxicity was identified for the substances lavendustin A, GF109203X, and rapamycin. Toxicity was concomitant with cell cycle arrest, but except HMGB1, no increased expression of immunogenic markers was identified with the combination treatment. The results were validated for GF109203X and rapamycin but not lavendustin A in the 3D model of different colorectal (HT29, SW480) and pancreatic cancer cell lines (MiaPaca, Panc01). In conclusion, our in vitro data suggest that combining oxidative stress with chemotherapy would be conceivable to enhance antitumor efficacy in HIPEC.

## 1. Introduction

Colorectal carcinoma is among the most common cancers in both men and women. Risk factors are smoking and obesity, besides a lack of exercise and a diet low in carbohydrates but rich in alcohol and red meat diet is to be named as a risk factor. Moreover, genetic predisposition may put some patients at risk [1,2]. The relative 5-year survival rates are 63% in men and 63% in women. However, the prognosis is highly dependent on the stage at diagnosis. Unfortunately, diffuse metastasis in the abdomen is often present before diagnosis [3,4]. This complication of peritoneal carcinomatosis (PC), in particular, is associated with inferior survival and is still challenging in its treatment [5,6]. Besides colorectal cancer, also other gastrointestinal cancers are able to diffusely metastasize and create peritoneal carcinomatosis. Especially the pancreas carcinoma is such a tremendous disease with meager survival rates [7,8,9]. Even with surgical R0 resection (a histologically confirmed complete removal of the primary tumor), auxiliary organ infiltrations can occur because tumor nodules are not easy to differentiate visually from the surrounding tissue [10]. In contrast to localized tumors, a variety of side effects, such as infiltration of the liver and lungs, blockage of the bile duct, or the pancreas, can arise in PC [11,12]. In addition to the attempt of surgical resection, current treatment regimens include radiation and HIPEC, intraperitoneal heat-induced chemotherapy [13]. Combinations of chemotherapeutic agents are often used. However, these two treatment approaches are associated with severe side effects. The chemotherapeutic agents are often dissolved in a sufficient volume of sodium chloride that is pumped into the peritoneum to flush the surgical site [14]. The heated fluid is absorbed through the peritoneum so that it can cause systemic side effects. In addition, many rapidly mutating tumors develop resistance to specific chemotherapeutic agents [15,16,17,18,19]. Hence, there is a need for new therapeutic avenues for the peritoneal lavage of patients suffering from PC.

Protein kinases are a group of enzymes that can reversibly transfer a phosphate group to the hydroxyl group of amino acids in proteins. Together with the counteracting protein phosphatases, they precisely alter protein function in many cellular processes [20]. Protein kinases can be subdivided into two major classes, which are either specific for the phosphorylation of Ser/Thr or tyrosine residues [21]. Approximately 2% of the human genome encodes for protein kinases with 518 different protein kinases known [22]. Besides membrane-bound receptors, which possess a glycosylated extracellular binding region linked to the cytosolic domain via a single hydrophobic transmembrane domain, there are also cytoplasmatic protein kinases [23]. Since protein kinases regulate a variety of signaling pathways, which are particularly vital for cell growth and proliferation [24], they are essential in physiology but also pathology, e.g., arteriosclerosis [25], diabetes [26], and precancerous lesions, as well as cancer [27,28,29,30,31]. For example, in cancer, a fusion of a receptor tyrosine kinase with a free tyrosine kinase leads to long-term oligomerization and activation, as in BCR-ABL fusion in chronic myeloid leukemia [32]. Furthermore, point mutations can lead to increased sensitivity to a ligand or even activation without a ligand, as in acute myeloid leukemia [33]. In some forms of breast cancer, there is overexpression of the receptor itself (HER-2/neu) [28].

Therefore the ‘kinome’, which is the complete set of protein kinases, has become an attractive target in cancer treatment with today 49 FDA-approved protein kinase inhibitors in clinical use. Small molecules modulate the catalytic activity by altering the binding of ATP or the kinases’ substrates. Antibodies can be directed against protein kinase ligands, their binding, or the receptor itself. Inhibiting the dimerization of the receptor tyrosine kinases, which is a crucial mechanism for their activation, can also be a target [27,30,31,34]. One specific target is epithelial growth factor receptor (EGFR), being responsible for cell proliferation, survival, motility, and cell cycle regulation. In many cancers—such as glioma, pancreatic carcinoma, ovarian cancer, breast cancer, small cell lung cancer, and up to 77% of colorectal cancers—EGFR is mutated and dysregulated [27,35]. After ligand binding, such as EGF or TGFβ, dimerization of the receptor and phosphorylation of tyrosine residues in the cytosolic domain occurs [36]. This phosphorylation allows several signal paths, such as the Ras/Raf/Erk, PI3K/Akt, PLC, and JAK/STAT signal path, to be activated [37]. In clinical trials, the anti-receptor antibodies cetuximab and panitumumab led to a better prognosis in 10% of patients with metastatic colorectal carcinoma [38]. This underlines the importance of such pathological changes and targeted pharmacological therapy [39]. Another target is protein kinase C (PKC) that is activated by growth factor-mediated phospholipase C (PLC) and is involved in many signaling processes. Downstream targets are mostly unknown, but the most important is thought to be Erk, GSK-3ß, NfκB, and the p-glycoprotein [40,41,42,43]. Hence, PKC is involved in many signaling pathways and therefore becomes interesting in its pathological upregulation and constant activation. For example, invasion and increased proliferation of intestinal cancer cells are associated with upregulation of PKCß [44,45,46]. Here, somatic mutations of the enzyme have been observed with the δ isotype being upregulated compared to non-malignant tissue [47]. Various PKC inhibitors have been tested in human phase I–III studies, but their efficiency has unfortunately been low which is likely due to limited bioavailability of the tested substances [48,49]. An additional target is Janus kinase 3 (Jak-3)which belongs to the *Janus* family of kinases and is most commonly expressed in hematopoietic but also in intestinal epithelial cells. Janus kinases are non-receptor tyrosine kinases, which are essential in the signal transduction of cytokine receptors as they have no intrinsical catalytic activity. In colorectal carcinoma, the dysregulation of JAK3 leads to increased invasion and progressive growth [50]. In 2012, the first JAK inhibitor, Ruxolitinib, was approved for the treatment of myelofibrosis and polycythemia vera so that further inhibitors are being investigated as potential treatment approaches in other types of cancer e.g., colorectal cancer [51,52,53]. Here, inhibition of JAK3 induced apoptosis and cell cycle arrest [54]. Metastasis and tumor growth in colorectal carcinoma is often promoted by a signaling pathway in which the mammalian target of rapamycin (mTOR) plays a crucial role. A mutation of mTOR is rarely found [55]. Therefore, dysregulation of this signaling pathway often is a cause of cancer [56]. In 23% of patients with colorectal carcinoma, a mutation of phosphatidylinositol-3-kinase (PI3K) can be detected [57]. This enzyme is negatively regulated by the phosphatase and tensin homologous (PTEN). If there is a mutation in the enzyme and too little PTEN activity, this results in increased activation of the tyrosine kinase mTOR [56]. Another regulatory step in mTOR activity is the Akt kinase. This kinase is, on the one hand, phosphorylated by the mTOR complex-2, but at the same time, regulates the activity of the mTOR complex-1 together with the PI3K and PTEN. All of these proteins were found in higher quantities in the context of colorectal carcinoma than in healthy tissue [58]. Increased mTOR activation leads to tumor growth [59], while mTOR inactivation reduces tumor growth in colorectal carcinoma [60,61]. Several mTOR inhibitors, like everolimus and temsirolimus, are used in treatment of breast cancer or renal cell carcinoma. Nevertheless, despite this great variety of inhibitors in cancer treatment, problems with drug resistance, reduced efficacy, and toxicity remain challenging in oncology [22]. Hence signaling pathways such as the mTOR pathway are also linked to reactive oxygen species (ROS). A combination of both could enhance the efficiency of specific protein kinase inhibitors [62].

ROS take part in crucial physiological cell functions, signaling pathways, and biochemical reactions. Non-malignant cells are in a balance of such reactions. This is mainly due to enzymes such as glutathione peroxidase and catalase, which can detoxify ROS [63,64]. For this study, we utilized low-dose hydrogen peroxide (50 µM H_2_O_2_) in a concentration where oxidative stress was induced without necrotizing cells. High concentrations are used clinically to disinfect skin or wounds at concentrations of 3% H_2_O_2_, which corresponds to 1 M. H_2_O_2_ is not an approved drug but notwithstanding a well-investigated molecule in cell and cancer biology. With low-dose H_2_O_2_, the antioxidant enzyme catalase is able to decompose H_2_O_2_ into water and oxygen [65,66,67]. Healthy cells contain about 10 nM H_2_O_2_ [68] and have a relatively high catalase activity. In contrast, many cancer cells have a 10 to 100-fold lower catalase activity [69]. The concentration of intracellular H_2_O_2_ depends primarily on the activity of this enzyme and the permeability of the cell membrane to extracellular H_2_O_2_ [63,70]. Compared to non-malignant cells, cancer cells often express more aquaporins through which H_2_O_2_ can enter [71]. At supra-physiological concentrations of H_2_O_2_, oxidation can damage DNA and lipids as well as denature, unfold, or alter the conformation of proteins and enzymes, thereby compromising their function. Dysfunctional proteins accumulate within the cell and cause stress, which can ultimately lead to cell death [66,72]. These mechanisms are generally summarized by the term cellular senescence [73]. Ultimately, these events can be fatal for cancer cells. For example, H_2_O_2_-enriching pharmaceuticals are under current investigation [74,75,76]. Moreover, the induction of oxidative stress is also one principle of photodynamic therapy (PDT) [77,78,79], ionizing radiation [80,81], or cold physical plasma [82,83]. Being a novel medical technology, medical plasmas utilize the generation of various ROS to tackle cancer, which was already successful in the treatment of patients with head and neck cancer [84,85]. Accordingly, several chemical and biological effects can be abrogated by adding ROS scavengers [86,87,88]. ROS-induced cancer cell death can also be highly immunogenic [89] (immunogenic cell death, ICD). Plasma treatment can induce ICD as well [90]. Specifically, cells subjected to ICD can foster antigen-specific immune responses against tumor-associated antigens and neoantigens [91,92,93]. This is preceded by activation of professional antigen-presenting cells via danger-associated molecular patterns (DAMPs) such as calreticulin (CRT), heat-shock protein 70 and 90 (HSP70, HSP90, and high-mobility group box 1 protein (HMGB1) [92]. Hence, ICD has significant potential in cancer therapy, in that tumors can be tackled that have previously escaped an endogenous immune response [94,95,96]. ICD may have a positive prognostic value [97] as a basis to generate T-cell responses, which are supported via checkpoint immunotherapy.

The aim of this study was to screen a protein kinase inhibitor library of 80 compounds against additive cytotoxicity with H_2_O_2_ in colorectal cancer cells with the vision to improve current HIPEC in patients suffering from PC. By studying several cytotoxicity and ICD parameters in 2D or 3D tumor models, we were able to identify two targets that may be potential candidates for such an approach.

## 2. Results

### 2.1. Screening a Library of 80 Different Kinase Inhibitors Identified Four Substances with Additive Toxicity in Combination with H_2_O_2_

**Figure 1:**
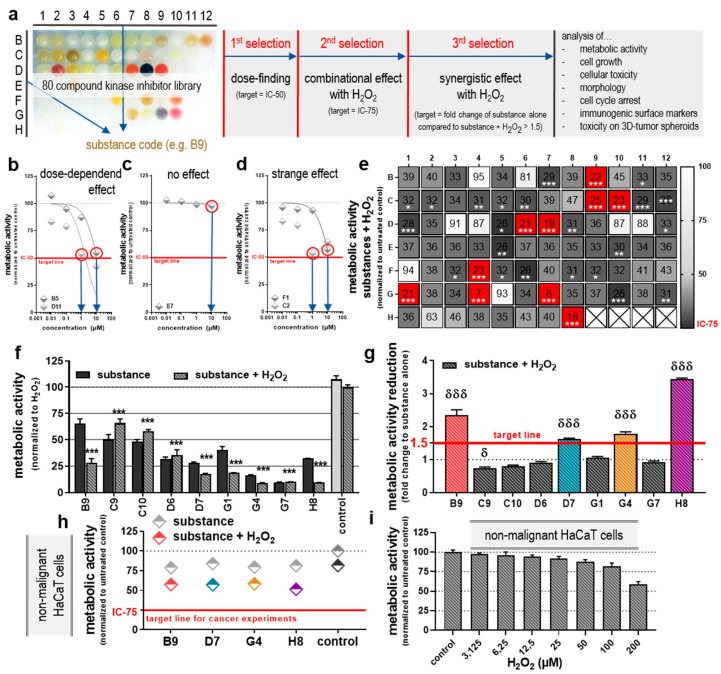
Experimental procedures and selection of kinase inhibitors. (**a**) Experimental strategies for the identification of selected kinase inhibitors with synergistic effect with H_2_O_2_ out of a 80 compound inhibitor library; representative drugs that show (**b**) dose-dependent, (**c**) no, or (**d**) an odd effect on the reduction of cancer cell metabolic activity 24 h post-incubation with the kinase inhibitors and the concentration that was chosen to reach 50% of reduction (blue arrows); (**e**) metabolic activity of cancer cells with its numerical value that had received kinase inhibitors together with H_2_O_2_ for 24 h (red: substances that reached 75% reduction in combination with H_2_O_2;_ crossed fields: no substances tested); (**f**) detail view of the cancer cell metabolic activity +SEM 24 h post-incubation with substances alone or in combination with H_2_O_2_; (**g**) increase of metabolic activity reduction +SEM of substances + H_2_O_2_ compared to substances alone, and selection (red line) of four inhibitors with a fold change of this increase >1.5 (colored bars); (**h**) metabolic activity of non-malignant HaCaT keratinocytes that received the four selected kinase inhibitors alone or in combination with H_2_O_2_; (**i**) metabolic activity of non-malignant HaCaT keratinocytes exposed to H_2_O_2_ in different concentrations. Significance levels for the comparison of substances without H_2_O_2_ to the respective substances with H_2_O_2_ (δ), and of their combination (with H_2_O_2_) to the H_2_O_2_-alone control (*) were determined via ANOVA. Data are representatives out of three (b–e) or two (f–i) independent replicates.

Kinase inhibitors are regularly used in the therapy of various cancers. In this study, 80 different substances were used to identify drugs that have significantly added toxicity upon the combination with low-dose H_2_O_2_ in vitro. During the experimental procedures, the most intoxicating substances were identified after three selection steps, and substance codes were used to analyze in a blinded fashion (Figure 1a). The code was kept for labeling graphs to enhance readability. The respective substances can be found in Table 1. Incubating CT26 colorectal cancer cells with tyrosine kinases either reduced the cells’ metabolic activity in a dose-dependent fashion (Figure 1b), did not reduce their metabolic activity (Figure 1c), or did not produce a clear dose–response relationship (Figure 1d). First, a specific dose for each substance was selected in this experiment, where the target was a 50% reduction compared to untreated control. Second, the substances were combined with low-dose H_2_O_2_ (50 µM), and the cancer cells’ metabolic activity was assessed 24 h later to identify additive cytotoxic effects (Figure 1e). The low-dose of H_2_O_2_ was used to induce oxidative stress to the cells in the absence of excessive cytotoxicity. In combination, some tyrosine kinase inhibitors were not superior to H_2_O_2_ alone, where others significantly reduced the metabolic activity in an additive to synergistic fashion. The sequence of treatment (first drug, then H_2_O_2_, or first H_2_O_2_, then drug) was found to be negligible (Supplementary Figure S1a–b). Subsequently, all inhibitors were chosen for further investigation (second selection) that led in combination with H_2_O_2_ to a >75% reduction of metabolic activity compared to H_2_O_2_ alone (for the concentrations, see Supplementary Table S1). These substances (B9, C9, C10, D7, G1, G4, G7, and H8) were investigated in more detail. In both settings, substances alone, and substances + H_2_O_2_, a reduction was observed for the tested colorectal cancer cells (Figure 1f). Calculating the fold change of mono vs. combination therapy, four substances (B9, D7, G4, and H8 at 100 µM) gave a more than 1.5-times higher reduction of the cancer cells metabolic activity compared to the substances alone (Figure 1g). Especially H8 developed a strong synergistic effect with H_2_O_2_ and was almost 4-times more effective than in monotherapy. Before further testing these four most interesting protein kinase inhibitors, its effect on non-malignant HaCaT keratinocytes was tested in combination with 50 µM H_2_O_2_. The combinational regimen reduced the metabolic activity of the non-malignant cells but did not reach the target line as it was applied for CT26 cancer cells (Figure 1h). Hence, if non-malignant cells would have been utilized for this screening, the four selected inhibitors would not have been identified as effective and would not have been selected. Additionally, to confirm that the utilized doses of H_2_O_2_ are semi-toxic, HaCaT keratinocytes were exposed to different concentrations of up to 200 µM. All tested concentrations induced only a mild reduction in the metabolic activity, while at twice (100 µM) of the concentration used for the screening, a 25% reduction was observed (Figure 1i).

### 2.2. Combination of Selected Kinase Inhibitors with H_2_O_2_ Reduced Cell Growth and Increased Cytotoxicity

**Figure 2:**
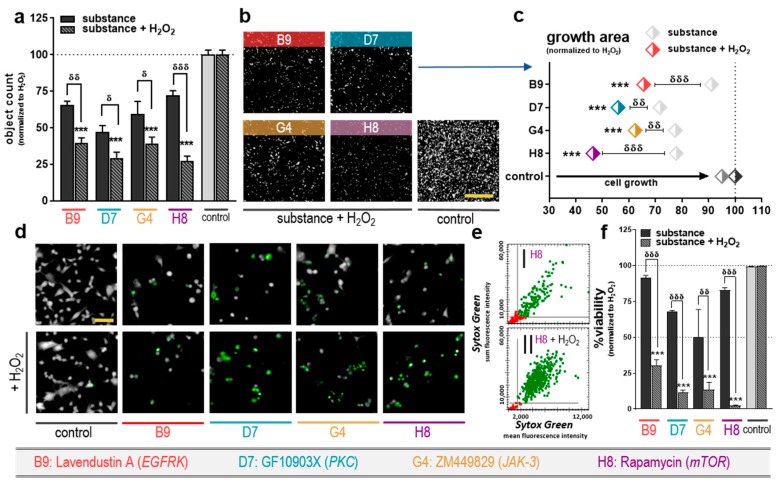
Combination of selected kinase inhibitors with H_2_O_2_ reduced cell growth and were cytotoxic in colorectal cancer cells. (**a**) Cell count +SEM from high content image analysis of cancer cells that received selected inhibitors (+/− H_2_O_2_) at 24 h. (**b**) Representative images of the cells’ cytosolic signal intensity (digital phase contrast) from nine fields of view (scale bar = 900 µm); (**c**) quantification of growth area; (**d**) representative images of the cells cytosolic signal and their Sytox Green fluorescence (scale bar = 150 µm) 24 h post-incubation with the selected substances (+/− H_2_O_2_); (**e**) representative selection strategy to determine Sytox Green^+^ (terminally dead) cells with imaging after treatment with substance H8 (+/− H_2_O_2_); (**f**) quantification of cell viability +SEM 24 h post-incubation with the substances. Significance levels for the comparison of substances without H_2_O_2_ to the respective substances with H_2_O_2_ (δ), and of their combination (with H_2_O_2_) to the H_2_O_2_-alone control (*) were determined via ANOVA. Data are representatives out of two independent replicates.

Assessment of metabolic activity is not equal to cell death nor cell proliferation. To analyze these cellular traits in more detail, the four previously selected kinase inhibitors (B9: lavendustin A; D7: GF109203X; G4: ZM449829; H8: rapamycin) were investigated regarding their potential of reducing cellular growth and increasing cytotoxicity. Twenty-four hours post-exposure, all four tested inhibitors significantly reduced the number of cancer cells if combined with H_2_O_2_ (Figure 2a). A similar effect was observed analyzing the cellular growth area of the cancer cells (Figure 2b), finding all substances combined with H_2_O_2_ to reduce the cytosolic area significantly compared to H_2_O_2_ or the substances alone (Figure 2c). The most substantial growth inhibition was observed for H8 + H_2_O_2_. To analyze terminal cell death, colorectal cancer cells were stained with Sytox (Figure 2d). Using algorithm-based object segmentation and analyzing each object’s Sytox intensity, dramatic terminal cell death was observed for the combination treatment and to a lesser extent, for drug monotherapy (Figure 2e). This suggested that the drug monotherapies are cytostatic, whereas the combination treatment was cytotoxic. H_2_O_2_ was used at a pre-determined low-dose concentration (50 µM), which did not induce terminal cell death in our treatment regimen (Figure 2f light grey bar). Hence, we conclude a synergistic cytotoxic effect for the combination therapy of tyrosine kinase inhibitors and low-dose H_2_O_2_. Another way to generate a plethora of reactive oxygen species, such as hydrogen peroxide, in parallel is the exposure of the cells to cold physical plasmas. Such plasmas, as induced by different devices as the kINPen or Plasma Soft Jet, lead to a time-dependent induction of H_2_O_2_ in the cell culture medium, which showed to reduce the metabolic activity of different cells (Supplementary Figure S2a–b). The treatment times that were needed for the deposition of 50 µM H_2_O_2_ did vary between the jets (43 s to 53 s) outlining their different structures. Moreover, these plasmas changed the morphology and growth of the cells similarly to H_2_O_2_ and can be considered for further use in similar combinations (Supplementary Figure S2c–e).

### 2.3. Combination of Selected Kinase Inhibitors with H_2_O_2_ Leads to Morphological Alterations, Cell Cycle Arrest, and Modulated Surface Marker Expression

**Figure 3:**
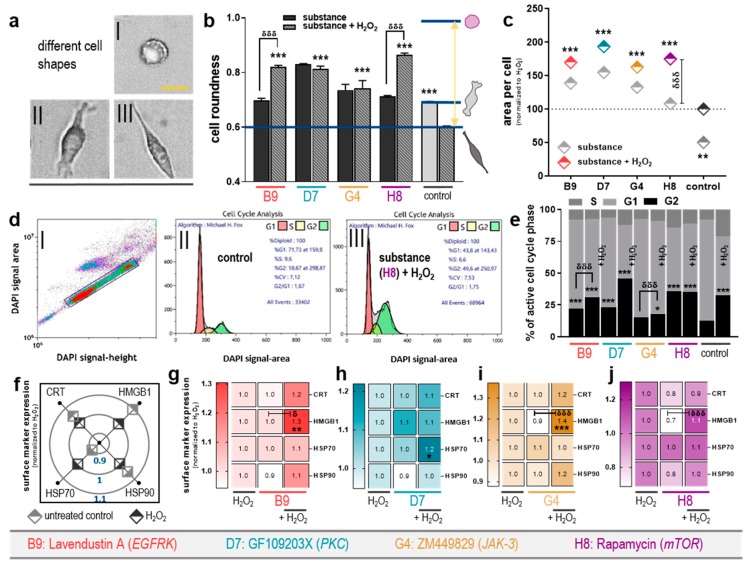
Combination of selected kinase inhibitors with H_2_O_2_ led to morphological alterations, cell cycle arrest, and modulated surface marker expression in colorectal cancer cells. (**a**) Representative brightfield images (scale bar = 50 µm) of colorectal cancer cells in a i) round, ii) spindle, or iii) elongated shape; (**b**) quantification of the cells’ roundness +SEM; and (**c**) area per cell 24 h post incubation with selected kinase inhibitors (+/− H_2_O_2_); (**d**) i) representative gating of DAPI signal (amount of nuclear acid) and the algorithm-based analysis of cell cycle phases of ii) untreated control cells, or ii) cells that received substance H8 + H_2_O_2_; (**e**) quantification of cell cycle phases and comparison of the percent of cells in the G2-phase; (**f**) modulation of the surface expression of calreticulin (CRT), high-mobility group box 1 protein (HMGB1), and the heat-shock proteins (HSPs) 70 and 90 in cells that were left untreated or received H_2_O_2_ alone for 24 h (inner ring fold change = 0.9; middle ring: no change; outer ring: fold change = 1.1); analysis of these surface markers on cancer cells that were incubated with the substances (**g**) B9, (**h**) D7, (**i**) G4, or (**j**) H8 with or without H_2_O_2_ for 24 h (blank fileds = decrease in marker expression). Significance levels for the comparison of substances without H_2_O_2_ to the respective substances with H_2_O_2_ (δ), and of their combination (with H_2_O_2_) to the H_2_O_2_-alone control (*) were determined via ANOVA. Data are representatives out of two independent replicates.

The effects on metabolic activity and viability may correlate with functional or morphological alterations in cancer cells. To address this, colorectal cancer cells treated with the four selected tyrosine kinase inhibitors (+/− H_2_O_2_), and detailed microscopic analysis of cellular morphology (Figure 3a) was performed. The treatment regimens introduced changes from spindle-like (untreated control) to elongated (H_2_O_2_ alone) and rounded shaped cells (drug mono and combination treatment) (Figure 3b). Surprisingly, adding H_2_O_2_ to the substances B9: lavendustin A and H8: rapamycin significantly increased their morphology towards shifting to a rounded cell type, whereas combination with D7: GF109203X and G4: ZM449829 did not produce such an effect (Figure 3b). The extent of such rounding may change in the kinetic of the post-treatment but when investigated at the same time point as all other assays still give valuable information on the cellular changes observed. In parallel, the single-cell area was quantified using algorithm-based image segmentation tools. Exposing the cells to H_2_O_2_ alone led to a significant decrease in individual cell size when compared to untreated control cells (Figure 3c). By contrast, an increase in individual cell area was observed upon incubation with the kinase inhibitors and in combination with H_2_O_2_. For combination treatment, this effect was enhanced in tendency compared to drug monotherapy (B9: lavendustin A, D7: GF109203X, and G4: ZM449829) and significantly for H8: rapamycin (Figure 3c). The results were obtained from more than 2000 individual cells segmented per condition. The increase in individual cells’ size can be a hallmark of cellular senescence and cell cycle arrest. Using nuclear acid staining and algorithm-based cell cycle phase analysis (Figure 3d), the number of colorectal cancer cells halted in the G2 phase was elevated in all treatment regimens, including H_2_O_2_ alone (Figure 3e). Combination vs. drug monotherapy gave an increase with all four drugs. To analyze the potential immunogenic consequences of the cytotoxic treatment regimens, multicolor flow cytometry was performed assessing the expression of several DAMPs important to elicit antitumor immunity. Compared to untreated cells and cells that were exposed to H_2_O_2_ alone, only modest modulation of cell surface markers was observed, ranging from 0.9- to 1.1-fold change (Figure 3f). Normalizing each combination treatment to H_2_O_2_ mono treatment (Figure 3g–j), significant upregulation was seen for HMGB1 in for all four combination treatments. HSP90 was increased in tendency for all for drug-H_2_O_2_ combination treatment when compared to drug alone. For CRT, a similar increase was observed, while HSP70 was only increased in tendency for D7: GF109203X.

### 2.4. Only Three Out of Four Selected Kinase Inhibitors Showed Enhanced Toxicity in Combination with H_2_O_2_ in 3D Tumor Spheroids

**Figure 4:**
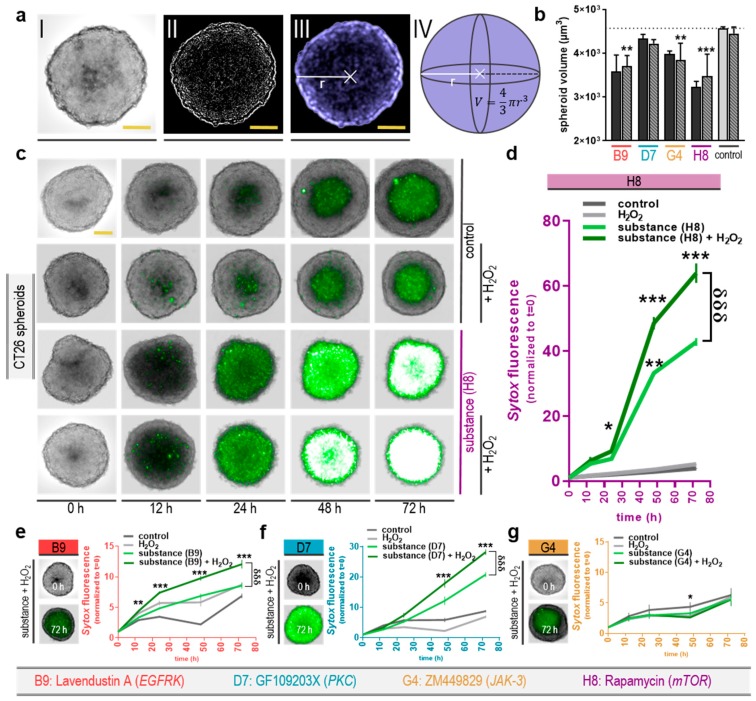
Toxicity of selected kinase inhibitors with H_2_O_2_ in 3D tumor cell spheroids of CT26 colorectal cancer cells. (**a**) Representative high-content image analysis strategy of 3D cancer spheroids shaped from initially 3 × 10^3^ cells shown as i) brightfield image (scale bar = 300 µm), ii) processed high contrasted image, with iii) detected spheroid area and iv) the volume calculation approach; (**b**) calculated spheroid volume +SEM 72 h post-incubation with selected kinase inhibitors (+/− H_2_O_2_); (**c**) representative maximum projection intensity images from 16 z-stacks in brightfield and Sytox Green fluorescence channel imaged over a 72 h time-course (scale bar = 300 µm) under incubation with the substance H8 under various conditions; (**d**) quantification of the Sytox mean fluorescence intensity +SEM inside the spheroid region during this time-course; representative images of selected kinase inhibitors with H_2_O_2_ at t = 0 and t = 72 h; (**e**) the quantification for the substances B9, (**f**) D7, and (**g**) G4 +SEM. Significance levels for the comparison of substances without H_2_O_2_ to the respective substances with H_2_O_2_ (δ), and of their combination (with H_2_O_2_) to the H_2_O_2_-alone control (*) were determined via ANOVA. Data are representatives out of three independent replicates.

Three-dimensional tumor cell models allow for more cellular heterogeneity and therefore are regarded as appropriate tools to further test novel antitumor approaches. To validate the combination treatments of the four compounds with H_2_O_2_ that were identified in 2D cultures, 3D colorectal cancer cell tumor spheroids from CT26 cells were generated. At several time points of exposure to mono or combination treatments, spheroids were imaged and analyzed using algorithm-based imaging tools (Figure 4a). A decrease in the spheroid volume was observed at 72 h post-treatment, which was significant for B9: lavendustin A, G4: ZM449829, and H8: rapamycin plus H_2_O_2_ (Figure 4b). Tracking cytotoxic effects in 3D tumor spheroids during the 72 h time-course revealed an increase of toxicity with increasing culture time, which was also observed in controls to a modest extent (Figure 4c). However, quantitative image analysis revealed a substantial (60-fold) increase in cytotoxicity in colorectal cancer cells that were incubated with H8: rapamycin + H_2_O_2_ (Figure 4d), which was significantly lower in the drug mono treatment group (40-fold), and negligible in control spheroids and spheroids receiving H_2_O_2_ alone (4-fold). Similar effects, although to a lesser extent, were observed for the kinase inhibitors B9: lavendustin A and D7: GF109203X (Figure 4e–f). Only substance G4: ZM449829 failed to induce a significant toxic effect in tumor spheroids in both mono and combination treatment (Figure 4g). A summary of the effects observed with all four tyrosine kinase inhibitors in combination with low-dose H_2_O_2_ is presented in Table 2.

### 2.5. Toxic Effects of Two of Our Three Selected Kinase Inhibitors Were Validated in a 3D Tumor Model of Different Colorectal and Pancreatic Cancer Cells.

**Figure 5:**
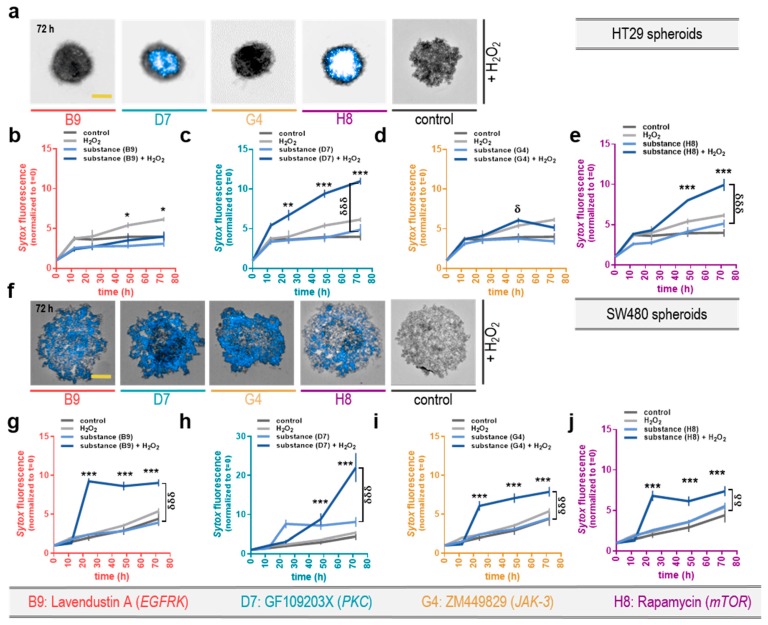
Validation of the toxicity of selected kinase inhibitors with H_2_O_2_ in 3D tumor cell spheroids of HT29 and SW480 colorectal cancer cells. (**a**) Representative maximum projection intensity from 16 z-stack images of spheroids formed from HT29 colorectal cancer cells (scale bar = 500 µm) and the quantification of the Sytox mean fluorescence intensity +SEM inside the spheroids shaped from initially 3 × 10^3^ cells during a 72 h time-course; (**b**) representative images of spheroids from HT29 colorectal cancer cells during a 72 h time course exposed to the substances B9, (**c**) D7, (**d**) G4, and (**e**) H8 (+/− H_2_O_2_); (**f**) representative maximum projection intensity images from 16 z-stacks of SW480 colorectal cancer cell spheroids (scale bar = 500 µm); (**g**) the quantification of the Sytox mean fluorescence intensity +SEM inside the spheroids after exposure to the substances B9, (**h**) D7, (**i**) G4, and (**j**) H8 (+/− H_2_O_2_) Significance levels for the comparison of substances without H_2_O_2_ to the respective substances with H_2_O_2_ (δ), and of their combination (with H_2_O_2_) to the H_2_O_2_-alone control (*) were determined via ANOVA. Data are representatives out of five (a–e) or three (f–j) independent replicates.

After identifying three kinase inhibitors to actively induce toxicity in tumor spheroids shaped from CT26 colorectal cancer cells, their anti-tumor capacity needed to be validated in another model of tumor spheroids. Applying the same treatment regimen to HT29 colorectal cancer cells in a 3D spheroid culture, increased the Sytox Blue dead cell stain intensity during a 72 h time-course. Especially the substances D7: GF109203X and H8: rapamycin, together with H_2_O_2_, again induced cell death that was significantly increased compared to H_2_O_2_ alone or their respective mono treatment (Figure 5c,e). However, changes were not as stable as seen before in CT26 spheroids, and B9: lavendustin A and G4: ZM449829 failed to increase the effect of H_2_O_2_ (as seen for B9: lavendustin A where H_2_O_2_ exceeds the combinational treatment, Figure 5b,d). Moreover, cell death was quantified in another colorectal cancer cell line, SW480 (Figure 5f). All three tested colorectal cancer cell lines responded significantly to the combination of D7: GF109203X and H8: rapamycin (Figure 5h,j). The SW480 cells were very vulnerable to the combination treatments with low-dose H_2_O_2_, and all combinational regimens were significant compared to the control at t = 72 h post-treatment, which was not found for the monotherapies with the inhibitors alone (Figure 5g–j). The best induction of cell death was observed for the inhibitor D7: GF109203X (Figure 5h). Interestingly, the SW480 spheroids reached a toxicity plateau at t = 24 h, which then fairly preserved the effect, except for D7: GF109203X, where even after 24 h the toxicity increased. As these promising findings should not be limited to one tumor entity that can induce peritoneal carcinomatosis, we further tested the most promising kinase-inhibitors on pancreatic cancer cell spheroids. Three-dimensional tumor spheroids formed from MiaPaca cells also responded to the treatment regimen in a time-dependent manner and showed increased Sytox signals after the 72 h time course (Supplementary Figure S3a). All in all the combinational regimen did not cause induction of toxicity as strong as for CT26 cancer spheroids, but all substances were significantly more effective in their combination with H_2_O_2_ than in their mono treatment (Supplementary Figure S3b–e). Additionally, the inhibitors B9: lavendustin A, G4: ZM449829, and H8: rapamycin was also significantly toxic compared to the control regimen (Supplementary Figure S3a). In contrast to the spheric structure of compact spheroids, Panc01 pancreatic cancer cells form only loose constructions. (Supplementary Figure S3f). After the exposure of these spheroids to the toxic compounds, destruction and fragmentation were observed. The combination of H_2_O_2_ and B9: lavendustin A, D7: GF109203X and H8: rapamycin significantly reduced the spheroids’ ‘compactness’ (morphology parameter that was calculated by the distribution of strong brightfield signals inside the spheroid region) compared to H_2_O_2_ in monotherapy (Supplementary Figure S3g). This parameter was chosen because of technical limitations in assaying terminally dead cells. Spheroids with many dead cells tend to ‘fall apart’ and by that increase the area and decrease the compactness score, which was quantitated in an unbiased manner using algorithm-driven image quantification. In every tested substance, this effect was higher in the combinational regimen and was significant for B9: lavendustin A and D7: GF109203X (Supplementary Figure S3g). As the second step, the area of this decomposed structure was quantified using the same software-based analysis tools, and it was found that this destruction is time-dependent and increased to greatest extend at t = 72 h but also during the whole time-course (as shown as AUC: Supplementary Figure S3h–k). All regimens in combination with G4: ZM449829 failed to induce toxicity in Panc01 spheroids, too, while B9: lavendustin A, D7: GF109203, and H8: rapamycin developed substantial and significant toxicity with H_2_O_2_. Interestingly, B9: lavendustin A and D7: GF109203X was significantly more effective in the combination as opposed to H8: rapamycin, which was effective in combinational and also in monotherapy (Supplementary Figure S3k). These findings suggested the potential of reactive species in combination with two selected kinase inhibitors (D7: GF109203X and H8: rapamycin) to enhance toxicity in three different (CT26, HT29, Panc01) 3D cancer spheroid models.

## 3. Discussion

The aim of this work was to identify protein kinase inhibitors that acted in an additive or synergistic manner with H_2_O_2_ (itself applied at low-dose, non-toxic conditions) cytotoxic against colorectal cancer cells. The hypothesis was that a combination of several stress pathways (blockage of growth signals together with oxidative stress) leads to additive cytotoxicity in tumor cells. Clinically, this might lead to a reduction of drug concentrations needed, resulting in a decrease of toxic side effects while at the same time having similar or even increased therapeutic efficacy. For this purpose, a compound library of 80 different inhibitors was tested in combination with low-dose H_2_O_2_ by assessing various parameters such as cellular metabolic activity, morphology, cell cycle arrest, immunogenic cell death (ICD), and cytotoxicity in 2D and 3D in vitro models.

The benefit of targeted therapy is to achieve the most toxic effect possible concerning the tumor cells without damaging the surrounding tissue and thus limiting the dose of the treatment [98]. However, one cause of the failure of cancer therapy is resistance to chemotherapy. Resistance is mediated by a high rate of mutations and can develop before or during treatment, i.e., exposure to the drugs [99]. In many treatment regimens, such as HIPEC, various chemotherapeutic agents are used in combination [12,14]. Ways to avoid resistance is the use of new chemotherapeutic agents and the combination with other treatment regimens such as oxidative stress [100] to damage the tumor cells using several pathways in parallel [101,102]. Especially in the treatment of peritoneal carcinomatosis, as a complication of colorectal carcinoma, our approach of using H_2_O_2_ seems promising since during HIPEC the abdominal cavity is flushed with heated chemotherapeutic agents [12] that only act locally but also offer seamless combination with agents other than chemotherapy alone.

Low-dose concentrations of ROS, as we used in our experiments, are able to interfere in various cellular signaling processes. Several redox-sensitive target molecules can be activated or inactivated by even mild changes in the intracellular redox state. On target molecule is thioredoxin (Trx), which, under normal conditions, inhibits the apoptosis signaling regulating kinase 1 (ASK1), also known as mitogen-activated protein kinase 5 (MAP3K5). Rising ROS concentrations induce Trx dimerization [103] and, therefore, dissociation of the ASK1/Trx complex leading to the activation of several downstream targets like MKK3/7, MKK4/MKK7, p38, and JNK which are part of the mitogen-activated protein kinase pathway [104,105,106,107]. As such, ASK-1 as well as its downstream targets, are crucial in a variety of cellular responses to oxidative stress, e.g., apoptosis, differentiation, and inflammation. Both p38 and JNK can also be directly activated after exposure to low micromolar hydrogen peroxide concentrations [108,109,110]. Another step in oxidative stress signaling is the ROS-mediated increase of cytosolic Ca^2+^ levels. Cytosolic Ca^2+^ plays a role in activation of several signaling paths including apoptotic processes [111]. Relatively small amounts of H_2_O_2_ are also able to induce the mRNA of the protein c-Fos and c-Jun which form the transcription factor AP-1 that is involved in cell growth, differentiation, and apoptosis [112]. Activation of the mentioned pathways might not be enough to promote a cytotoxic response after treatment, because they are involved in the complex regulation system of several interfering signaling processes. Nevertheless, our results show that the combination of both oxidative stress (low-dose H_2_O_2_) and drug-induced blockage of growth signaling pathways enhance each other’s property to induce apoptosis in tumor cells. We identified three protein kinase inhibitors that, in combination with low-dose H_2_O_2_, showed synergistic toxicity (terminally dead cells) in colorectal carcinoma cells (CT26) in 2D and 3D tumor models. These substances were B9: lavendustin A, D7: GF109203X, (G4: ZM449829 but only in 2D cultures), and H8: rapamycin. The effectiveness of two of these substances, GF109203X and rapamycin, was validated in a 3D tumor model of HT29 and SW480 colorectal, as well as Panc01 pancreatic cancer cells (while D7: GF109203X failed to induce constant significant changes in MiaPaca pancreatic cancer cells). The most potent toxic effect was found in the combination of H_2_O_2_ with the substance H8: rapamycin, the weakest with B9: lavendustin A. Our results indicated differences across several assays worth mentioning. For example, addressing metabolic activity cannot differentiate between cytostatic and cytotoxic effects, and it was interesting to note that the drugs alone were cytostatic (which was also observed in assaying cell area and cell cycle phases), while drugs combined with low-dose H_2_O_2_ were cytotoxic. In oncology, a combination of both effects is desired as both reduced proliferation [113], as well as cytotoxic effects, contribute to tumor control [114]. This is of added value as many tumor cells acquire mutations to circumvent such an arrest [115]. Moreover, oxidation of intracellular proteins, such as H_2_O_2_, can lead to such persistence in the G2 phase [116]. These oxidized proteins and the arrest of the cell cycle are central features of cellular senescence [72,73] and may work in concert with chemotherapeutics [117]. Oxidative reactions within the cell not only lead to direct protein modifications but also act on redox signaling pathways [118] that regulate, for instance, rapid remodeling of the cytoskeleton [119,120]. Concordant with that, we observed characteristic morphological changes in the different treatment regimens. Exposure to H_2_O_2_ alone provoked a slightly elongated phenotype in cancer cells. As subtoxic concentrations of ROS can stimulate cellular proliferation this could speak for a more mesenchymal-like phenotype of the cells [121]. Contrarily, the combinational treatment of H_2_O_2_ with different protein kinase inhibitors showed a more rounded morphology of the cells. As already mentioned, together with our observation of decreased cell growth and increase in individual cell size this could indicate cellular senescence [122].

A valuable tool to assess novel combination treatment avenues is the use of 3D tumor cell spheroids [123]. In such structures, tumor cells show a more regulated proliferation and differentiation behavior [124,125]. Similar to tumor nodules, spheroids can cause central necrosis by rapidly growing tumor cells [126]. To what extent a substance is effective against spheroids does not only depend on its intrinsic cytotoxic effect but also on its potential to diffuse into deep cell layers [127]. Thus, for example, the kinase inhibitor G4: ZM449829 neither showed a cytotoxic effect in combination with H_2_O_2_ nor when used alone, although substantial toxicity was observed in 2D cell culture experiments. The D7: GF109203 and in particular H8: rapamycin showed a robust toxic effect in the 3D tumor spheroids, which was also significantly increased in combination with H_2_O_2_ compared to single treatment with the substance. These results seem promising for testing such a combination regimen further, despite the lack of immunogenic features of our treatment. Engagement of antitumor immunity is an approach receiving increasing interest in oncology [128]. An anti-tumor immune response evoked in the context of ICD via DAMP release contributing to the maturation of dendritic cells [129,130] may lead to an improvement in the prognosis of various cancers [97]. Even though pro-oxidative therapies are capable of inducing ICD [131,132,133], we did not find an ICD signature common to all four combinations of protein kinase inhibitors with H_2_O_2_. The reason for this could be that the oxidative stress-mediated by H_2_O_2_ alone was cytostatic but not cytotoxic, while ICD requires the cells to die upon treatment [134].

Chemotherapeutic agents most commonly used in HIPEC are doxorubicin, mitomycin-C, cisplatin, as well as combinations of cisplatin and mephedrone [135]. One aim of this work was to identify other substances, which may add efficacy to the HIPEC treatment regime. Of these substances, B9: lavendustin A, a non-competitive inhibitor of the ATP binding pocket of the EGFR kinase [136], has already shown toxic effects on other types of tumor cells [137]. Moreover, Lavendustin A reduced neovascularization in a rat model, potentially reducing the engraftment of new vessels in tumors [138]. For D7: GF109203X, an inhibitor of protein kinase C, reduced migration and invasion of lung carcinoma cells was reported [139,140]. However, the same substance showed an increased proliferation of endometrial cancer [141]. The substance G4: ZM449829, a Jak3 inhibitor that failed to give cytotoxic effects in the 3D colorectal cancer model, may lead to immunosuppression due to reduced T cell proliferation [142,143]. The toxic tumor effect of this substance on various cancer cells is currently being investigated in other screenings campaigns [144,145]. A well-investigated drug inhibiting mTOR complex I is H8: rapamycin. This substance performed best in our study. It is not only used as anticancer drug but also to avoid graft rejection due to its immunosuppressive effect that may be promoted due to its increase of regulatory T-cells and increased sensitivity towards apoptosis of effector T-cells [146,147]. This fact may also explain the limited immunogenicity conferred either alone or in combination with low-dose H_2_O_2_ as observed in our study. mTOR affects a variety of signaling pathways, making it one of the most important regulators of cell growth [148]. Rapamycin has already been shown to have potent toxic effect on various types of tumor cells [149,150,151], as well as in colorectal carcinoma [152]. Notably, this substance is already being used as a chemotherapeutic agent in HIPEC [153,154]. However, its application is often limited by resistances to rapamycin [146,155].

## 4. Materials and Methods

### 4.1. Cell Cultivation

The colorectal carcinoma cells (CT26), derived from murine fibroblasts, were cultivated in Roswell Park Memorial Insitute (RPMI) 1640 medium (PanBiotech, Aidenbach, Germany), containing 10% fetal calf serum, 2% penicillin-streptomycin, and 2% glutamine (all Sigma-Aldrich, St. Louis, MI, USA). The same culture medium was applied to the human colorectal adenocarcinoma cell line, SW40. The human HT29 adenocarcinoma cells and human MiaPaca and Panc01 pancreatic epitheloid carcinoma cells were cultured in Dulbecco’s modified Eagle medium (DMEM; ThermoFisher, Waltham, MA, USA), containing equal supplements as described for RPMI. Cell splitting was performed regularly twice a week using phosphate-buffered saline (PBS), accutase (BioLegend, London, United Kingdom), and a cell culture incubator (Binder, Neckarsulm, Germany) at 37° C, 5% CO_2_, and 95% humidity. For counting live cells, cells were stained with PI and quantitatively measured via an acoustically focused flow cytometer (attune; ThermoFisher). For experiments with 2D-cell cultures, 1 × 10^4^ cells were seeded in 96° cell culture plates (Eppendorf, Hamburg, Germany) containing a rim that was filled with double-distilled water for enhanced evaporation protection of the outer wells (edge effect). To from 3D-tumor spheroids, 3 × 10^3^ cells were seeded in ultra-low attachment plates (PerkinElmer, Hamburg, Germany). For flow cytometry experiments, 1 × 10^5^ cells were seeded in 24-well cell culture plates with a water-filled rim to protect from edge effects (Eppendorf). Cells were incubated 24 h before they were utilized for further experimental processing.

### 4.2. Treatment Regimen

The 80 different protein kinase inhibitors were from a compound library (Enzo, Farmingdale, NY, USA) that was stored at −80 °C. To avoid repetitive freeze-thawing cycles, the samples were aliquoted to their working concentration and stored in separate working plates, which were thawed immediately before utilizing them for downstream assays. For treating the cells, the cell culture medium was removed and replaced with either 50 µM H_2_O_2_ or with the different inhibitors at their specific concentration for 15 min. Subsequently, the complementary treatment solution (either 50 µM H_2_O_2_ or the substances) were added to the cells for 24 h. Through this procedure, it was tested if cells behave differently upon receiving H_2_O_2_ either before or after the initial exposure to the kinase-inhibitors.

### 4.3. Plasma Treatment

To outline cold physical plasma as another method to induce hydrogen peroxide plasma treatment was performed using the kINPen (neoplas) and Plasma Soft Jet (engineered at the Plasma Bioscience Research Center, PBRC, Seoul, South Korea) argon plasma jets. These devices operate with 99.999% argon gas (Air Liquide, Paris, France), at two standard liters per minute. During the treatment, the gas flow, hight of the jets, and driving properties were standardized via an xyz-table (CNC-step, Geldern, Germany). The treatment of the cells was carried out in 96-well plates with 100 µL cell culture medium. The same amount of medium was used to quantify the levels of deposited hydrogen peroxide in the liquid post-treatment using the Amplex Ultra Red (ThermoFisher) assay according to the manufacturers’ instructions.

### 4.4. Metabolic Activity

After receiving the different substances from the kinase-inhibitor library (+/− H_2_O_2_), the cells were stored at optimal growing conditions for 24 h hours before resazurin (Alfa Aesar, Haverhill, MA, USA) was added at a final concentration of 100 µM. This metabolite can be converted by viable active cells to the fluorescent resorufin. The fluorescence of resorufin was then quantified using a multiplate reader (Tecan, Männedorf, Switzerland) at λ_ex_ 560 nm and λ_em_ 590 nm. Normalization was performed depending on the experimental question to either the untreated control or H_2_O_2_ alone.

### 4.5. Quantitative High Content Imaging Analysis

To assess the cytotoxicity of the various treatment regimens in more detail, analysis by high content imaging microscopy was performed. For this, the cells were incubated with 2.5 µM of a Sytox Green or Blue (ThermoFisher) solution for 10 min at 37 °C. Sytox Green and Blue binds to nuclear acids of dead cells that have lost their membrane integrity. Image acquisition was made using the high content/high throughput-imaging device Operetta CLS (PerkinElmer). The device acquires images with a 4.7-megapixel 16-bit sCMOS camera and using laser-based autofocus for high precision planarity. For the investigation of cell viability and morphology in 96-well plates, a 20x air objective (NA = 0.4; Zeiss, Jena, Germany) was used. For the microscopy of spheroids, a 5× air objective (NA = 0.16, Zeiss) was used. In the 96-well plates, several single images were taken per well in at least nine fields of view (FOVs). Different excitation LEDs were used to capture brightfield (BF) and digital-phase-contrast (DPC) images. For fluorescence microscopy, a λ_ex_ 475 nm LED with a λ_em_ 525 ± 25 nm bandpass filter or a λ_ex_ 405 nm LED with a λ_em_ 465 ± 35 nm bandpass filter was used to measure Sytox Green and Sytox Blue intensity levels. In preliminary experiments, all image acquisitions were optimized in favor of a well-focused image and optimum signal-to-noise ratio, and a standardized recording setup was created to assure precise imaging experiments across longitudinal experiments. Both the measurement and the subsequent analysis of the images were performed using the Harmony 4.9 (PerkinElmer) setup and analysis software. The evaluation strategy of the Sytox stain included a combination of the captured images of the fluorescence channel and the DPC signal, which reflects the pseudo-cytosolic cell surface. The latter allows for the segmentation of cells. At least 500 cells were segmented per well, and approximately 2000 single wells were analyzed in this study. Specifically, a combined image from both channels was used by the software segmenting objects for parameters such as intensity threshold and individual threshold, size and coefficients for sharing different signals. This was preceded by filtering the image by a sliding parabola function to increase the contrast and hence, segmentation accuracy. Within the detected cells of each well, the intensity of the fluorescence signal and the percentage of dead cells was calculated. Here, cells with relative mean fluorescence intensity (MFI) of greater than 1500 fluorescence units and a sum intensity greater than 5000 fluorescence units were considered as dead cells. Furthermore, various morphological parameters were calculated. All image quantification strategies were completely algorithm-based without the possibility of user intervention with regard to segmentation of, e.g., individual cells. Extended measurements were performed at 37 °C and 5% CO_2_ (live cell imaging) to avoid the toxic effects of unsuitable environmental conditions.

### 4.6. Flow Cytometry

To determine surface markers of ICD, 1 × 10^5^ CT26 cells were seeded in 1 mL fully supplemented cell culture medium in 24-well cell culture plates and incubated for 24 h. Subsequently, the cell culture medium was incubated with the different protein kinase inhibitors alone or in combination with H_2_O_2_ for another 24 h. Subsequently, cells were washed with 1 mL of PBS, and incubated with an antibody mix targeted against HSP70 conjugated to Alexa Fluor (AF) 488 (Abcam, Cambridge, United Kingdom), CRT (Novus, Littleton, CO, USA) conjugated to allophycocyanin (APC), HSP70 conjugated to AF700 (Novus), HMGB1 (BioLegend) conjugated to phycoerythrin (PE), the nucleic acid stain 4′,6-Diamidino-2-phenylindole (DAPI; BioLegend), and the accutase. Cells were incubated for 30 min at 37 °C before transferring the cell suspension to a 96 well v-bottom plate (Eppendorf). After centrifugation at 500× *g* for 5 min, cells were resuspended in PBS and washed twice before being taken up in a final volume of 75 μL per well. Individual cells of the suspension were acquired with a 4-laser flow cytometer equipped with a plate loader autosampler (CytoFLEX S; Beckman-Coulter, Brea, CA, USA) using CytExpert 2.0 software (Beckman-Coulter) as acquisition software. Forward scatter (FS) and side scatter (SSC), as well as the fluorescence of AF488, PE, APC, AF700, and DAPI was collected using specific band-bass filters. The spillover matrix was pre-determined using single-stained cells and setup beads (ThermoFisher). For analysis, the geometric mean values for each fluorochrome was determined in the live cell population, and exported for statistical evaluation. To analyze the different cell cycle phases, cells were treated and incubated as described above. After incubation, cells were detached, washed in PBS, and fixed with −20 °C ethanol for one hour. Subsequently, cells were washed and incubated with DAPI (final concentration 10 µM) for 30 min at 37 °C. After two further washes, single cells were acquired using flow cytometry. Analysis of FCS-files was performed using Kaluza 2.1 analysis software (Beckman-Coulter). This software contains a plug-in that allows determining cell cycle phases via an algorithm (Fox), overcoming the limitations of manual gating of cell cycle phases.

### 4.7. 3D Tumor Spheroids

To form three-dimensional tumor cell spheroids, 3 × 10^3^ CT26, HT29, SW480, MiaPaca, or Panc01 cells were seeded in 150 μL fully supplemented cell culture medium in 96-well plates (ThermoFisher) that prevent adhesion of cells by a special coating. Immediately after seeding, the suspension was centrifuged at 1000× *g* for 10 min to force an accumulation of cells in the center of the round bottom well. After incubation for 72 h, 125 μL of the cell culture medium was carefully removed and replaced with 50 μL of the protein kinase inhibitors in the corresponding concentration. After 15 min of incubation under cell culture conditions, 50 μL of a 50 μM H_2_O_2_ solution containing 2.5 μM of Sytox Green or Blue (with both stain nucleic acids as an indicator for cell death) was added. Immediately after that, as well as after 12 h, 24 h, 48 h, and 72 h of incubation, all spheroids were sequentially examined using high content imaging. The settings of the measurements were also standardized using a previously established measurement template. For image acquisition, taking into account the three-dimensional structure of such tumor cell nodules, 15 z-plane images (distance between planes: 5 µm) were acquired. For analysis, maximum intensity projections (MIP) were calculated for each spheroid to give 2.5D images. For enhanced contrasting and delimiting the spheroid from the background, a sliding parabola function was used. Subsequently, intensities of the Sytox fluorescence channel as well as morphological parameters were calculated using algorithm-based quantitative imaging tools. The morphology parameter “compactness” was also calculated using a morphology tool (STAR-morphology) that is provided by the Harmony 4.9 (PerkinElmer) analysis software. This utilizes the distribution of signals inside the brightfield channel (that absorbs more light than 60% of the average spheroid signal = compact regions) in comparison to the border of the spheroid region. Such compact spheroids get a high ‘compactness’ value, while this is low for spheroids composed of loose cells.

### 4.8. Statistical Analysis

Graphing and statistical evaluation were done using Prism 8.2 (GraphPad software, San Diego, CA, USA). Unless otherwise indicated, mean or standard error (SEM) is shown on the graphs. Mean values were obtained from individual data points of technical and biological replicates for the experiments on viability (resazurin assay) or from measurements for approximately 1 × 10^4^ individual cells in FACS or 5–10 × 10^3^ cells per well in high content imaging experiments. To avoid the accumulation of the α-error, one-way analysis of variance (ANOVA) or Kruskal–Wallis test was used as a non-parametric alternative for the statistical analysis comparing more than two groups. If several conditions were compared within several groups, a multi-factorial analysis of variance (ANOVA II) was used. Post-doc testing was done using Dunnett’s test. Trends were considered significant from the 95% confidence interval. Levels of significance were given as follows: * α = 0.05; ** α = 0.01; *** α = 0.001.

## 5. Conclusions

Tyrosine kinase inhibitors are potent anticancer drugs, but chemoresistance may limit its use. Targeting both tyrosine kinase activity and oxidative stress pathways simultaneously, we identified two potent combinations that led to synergistic toxicity in 2D and 3D colorectal cell culture models. The most effective combination was low-dose H_2_O_2_ together with rapamycin. As this drug is already utilized in HIPEC targeting diffuse colorectal peritoneal carcinomatosis, such an approach may complement existing therapies of colorectal cancer.

## Figures and Tables

**Table 1:** Overview of the kinase inhibitors utilized in this study. Listed are all inhibitors used in this study, their substance code used throughout the figures and text, the target structure of the inhibitors, and the CAS number.

Substance Code	Name	Kinase Target	CAS
B1	PD-98059	MEK	167869-21-8
B2	U-0126	MEK	109511-58-2
B3	SB-203580	p38 MAPK	152121-47-6
B4	H-7·2HCl	PKA, PKG, MLCK, PKC	84477-87-2
B5	H-9·HCl	PKA, PKG, MLCK, PKC	116970-50-4
B6	Staurosporine	Pan-specific	62996-74-1
B7	AG-494	EGFRK, PDGFRK	133550-35-3
B8	AG-825	HER1-2	149092-50-2
B9	Lavendustin A	EGFRK	125697-92-9
B10	RG-1462	EGFRK	136831-49-7
B11	TYRPHOSTIN 23	EGFRK	118409-57-7
B12	TYRPHOSTIN 25	EGFRK	118409-58-8
C1	TYRPHOSTIN 46	EGFRK, PDGFRK	122520-85-8
C2	TYRPHOSTIN 47	EGFRK	122520-86-9
C3	TYRPHOSTIN 51	EGFRK	122520-90-5
C4	TYRPHOSTIN 1	Negative control for tyrosine kinase inhibitors	2826-26-8
C5	TYRPHOSTIN AG 1288	Tyrosine kinases	116313-73-6
C6	TYRPHOSTIN AG 1478	EGFRK	175178-82-2
C7	TYRPHOSTIN AG 1295	Tyrosine kinases	71897-07-9
C8	TYRPHOSTIN 9	PDGFRK	10537-47-0
C9	Hydroxy-2-naphthalenylmethylphosphonic acid	IRK	120943-99-9
C10	PKC-412	PKC inhibitor	120685-11-2
C11	Piceatannol	Syk	10083-24-6
C12	PP1	Src family	172889-26-8
D1	AG-490	JAK-2	133550-30-8
D2	AG-126	IRAK	118409-62-4
D3	AG-370	PDGFRK	134036-53-6
D4	AG-879	NGFRK	148741-30-4
D5	LY 294002	PI 3-K	154447-36-6
D6	Wortmannin	PI 3-K	19545-26-7
D7	GF 109203X	PKC	133052-90-1
D8	Hypericin	PKC	548-04-9
D9	Ro 31-8220 mesylate	PKC	138489-18-6
D10	D-erythro-sphingosine	PKC	123-78-4
D11	H-89·2HCl	PKA	127243-85-0
D12	H-8	PKA, PKG	84478-11-5
E1	HA-1004·HCl	PKA, PKG	92564-34-6
E2	HA-1077·2HCl	PKA, PKG	103745-39-7
E3	2-Hydroxy-5-(2,5-dihydroxybenzylamino)benzoic acid	EGFRK, CaMK II	125697-93-0
E4	KN-62	CaMK II	127191-97-3
E5	KN-93	CaMK II	139298-40-1
E6	ML-7·HCl	MLCK	109376-83-2
E7	ML-9·HCl	MLCK	105637-50-1
E8	2-aminopurine	p58 PITSLRE β1	452-06-2
E9	N9-isopropyl-olomoucine	CDK	158982-15-1
E10	Olomoucine	CDK	101622-51-9
E11	Iso-olomoucine	Negative control for Olomoucine	101622-50-8
E12	Roscovitine	CDK	186692-46-6
F1	5-iodotubericidin	ERK2, adenosine kinase, CK1, CK2,	24386-93-4
F2	LFM-A13	BTK	62004-35-7
F3	SB-202190	p38 MAPK	152121-30-7
F4	PP2	Src family	172889-27-9
F5	ZM 336372	cRAF	208260-29-1
F6	SU 4312	Flk1	5812-07-7
F7	AG-1296	PDGFRK	146535-11-7
F8	GW 5074	cRAF	220904-83-6
F9	Palmitoyl-DL-carnitine	PKC	6865-14-1
F10	Rottlerin	PKCΔ	82-08-6
F11	Genistein	Tyrosine kinases	446-72-0
F12	Daidzein	Negative control for Genistein	486-66-8
G1	Erbstatin analogue	EGFRK	63177-57-1
G2	Quercetin·2H2O	PI 3-K	6151-25-3
G3	SU1498	Flk1	168835-82-3
G4	ZM 449829	JAK-3	4452-06-6
G5	BAY 11-7082	IKK signaling pathway	195462-67-7
G6	5,6-dichloro-1-β-D-ribofuranosylbenzimidazole	CK II	53-85-0
G7	2,2′,3,3′,4,4′-hexahydroxy-1,1′-biphenyl-6,6′-dimethanol dimethyl ether	PKCα, PKCγ	154675-18-0
G8	SP 600125	JNK	129-56-6
G9	Indirubin	GSK-3β, CDK5	479-41-4
G10	Indirubin-3′-monooxime	GSK-3β	160807-49-8
G11	Y-27632·2HCl	ROCK	146986-50-7
G12	Kenpaullone	GSK-3 β	142273-20-9
H1	Terreic acid	BTK	121-40-4
H2	Triciribine	Akt signaling pathway	35943-35-2
H3	BML-257	Akt	32387-96-5
H4	SC-514	IKK2	354812-17-2
H5	BML-259	Cdk5/p25	267654-00-2
H6	Apigenin	CK-II	520-36-5
H7	BML-265	EGFRK	28860-95-9
H8	Rapamycin	mTOR	53123-88-9

**Table 2:** Summary table of the key results identified in this study. Shown are the toxicity in 2D and 3D cultures with the combination treatment of the four in 2D cultures identified drugs, as well as cell cycle arrest and immunogenic cancer cell death-associated expression of cell surface markers. Effect intensity was graded (+++ = high, ++ = intermediate, + = modest, - = no effect).

Parameter	B9: Lavendustin A	D7: GF109203X	G4: ZM449829	H8: Rapamycin
Toxicity (2D)	+	++	++	+++
Toxicity (3D)	+	++	-	+++
Cell cycle arrest	+	++	-	+
ICD	+	+	+	-

## Supplementary Materials

The following are available online at https://www.mdpi.com/2072-6694/12/1/122/s1, Figure S1: The sequence of combination treatment was not significant.; Figure S2: Generation of hydrogen peroxide through cold-physical plasma jets introduces metabolic and morphological rearrangements; Figure S3: Validation of the toxicity of selected kinase inhibitors with H_2_O_2_ in 3D tumor cell spheroids of MiaPaca and Panc01 pancreatic cancer cells; Table S1: Concentrations of kinase inhibitors utilized in this study.
