# Supplementary material for: Identification of Two Kinase Inhibitors with Synergistic Toxicity with Low-Dose Hydrogen Peroxide in Colorectal Cancer Cells In vitro
Source: Cancers (Basel). 2020 Jan 2;12(1):122. doi: 10.3390/cancers12010122 (PMC7016670; doi:10.3390/cancers12010122)
Supplement: Supplementary file 1 [file cancers-12-00122-s001.pdf]

cancers

MDPI

Article

Identification of Two Kinase Inhibitors with Synergistic Toxicity with Low-Dose Hydrogen Peroxide in Colorectal Cancer Cells in vitro

Eric Freund, Kim Rouven Liedtke, Lea Miebach, Kristian Wende, Amanda Heidecke, Nagendra Kumar Kaushik, Eun Ha Choi, Lars-Ivo Partecke, Sander Bekeschus

Supplementary Materials

Supplementary Table 1: Concentrations of kinase inhibitors utilized in this study. Shown is the code of the different inhibitors and their identified concentration (conc. in µM) that was identified in the first experiments and used continuously in this study.

code	conc.	code	conc.	code	conc.	code	conc.	code	conc.	code	conc.	code	conc.
B1	100	C1	1	D1	100	E1	100	F1	10	G1	0.01	H1	100
B2	100	C2	1	D2	100	E2	100	F2	100	G2	100	H2	0.01
B3	100	C3	100	D3	0.01	E3	100	F3	100	G3	100	H3	100
B4	1	C4	100	D4	0.01	E4	100	F4	100	G4	100	H4	100
B5	10	C5	100	D5	100	E5	100	F5	100	G5	100	H5	100
B6	0,01	C6	10	D6	100	E6	100	F6	100	G6	100	H6	100
B7	10	C7	10	D7	100	E7	100	F7	100	G7	100	H7	100
B8	100	C8	1	D8	10	E8	100	F8	100	G8	100	H8	100
B9	100	C9	10	D9	0.1	E9	100	F9	100	G9	100		
B10	100	C10	100	D10	1	E10	100	F10	100	G10	100		
B11	10	C11	100	D11	1	E11	100	F11	100	G11	100		
B12	1	C12	100	D12	100	E12	100	F12	100	G12	100		

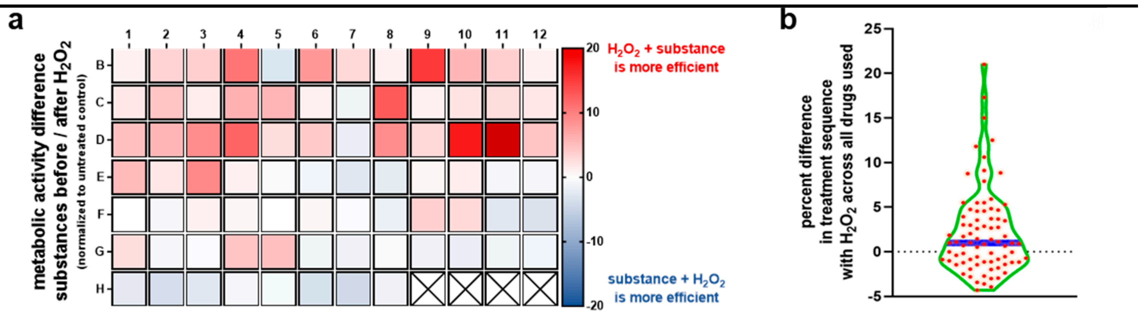

Supplementary Figure S1: The sequence of combination treatment was not significant. (**a**) Differences of metabolic activity reduction in colorectal cancer cells that were incubated with different kinase inhibitors and H_2O_2 either primed with H_2O_2 alone for 15 minutes (red fields) or received the substances 15 minutes before adding H_2O_2 (blue fields) displayed as a heat map; (**b**) same differences shown as violin scatter plot with single-cell events and median. The mean difference across all drugs was +2.18 % (median: 1.00 %, blue line), meaning that, in tendency, H_2O_2 sensitized for drug toxicity but only to a negligible extended.

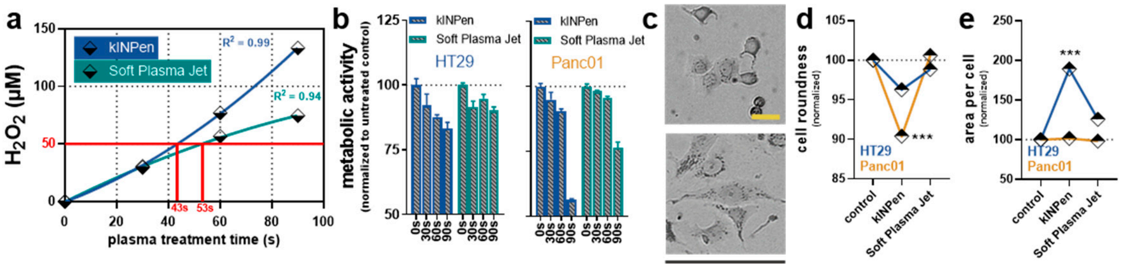

Supplementary Figure S2: Generation of hydrogen peroxide through cold-physical plasma jets introduces metabolic and morphological rearrangements. (**a**) Time-dependent induction of H_2O_2 in cell culture medium through the *kINPen* (INP) and *Soft Plasma Jet* (PBRC); (**b**) reduction in metabolic activity +SEM 4h post plasma-exposure with different sensitivities of HT29 colorectal and Panc01 pancreatic cancer cells; (**c**) distinct morphology of both cells types (scale bar = 30 µm); (**d**) cell roundness post-treatment and (**e**) calculated area per cell. Data are representatives out of three independent replicates.

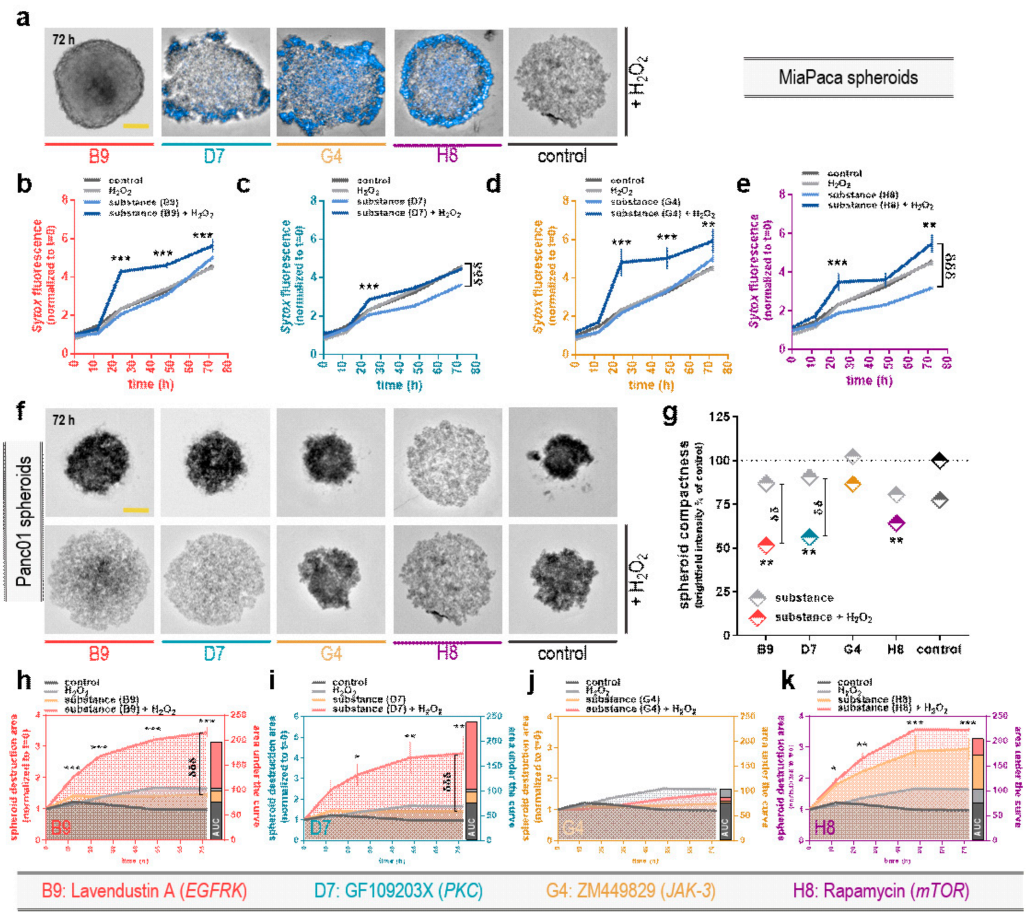

Supplementary Figure S3: Validation of the toxicity of selected kinase inhibitors with H_2O_2 in 3D tumor cell spheroids of MiaPaca and Panc01 pancreatic cancer cells. (**a**) Representative maximum projection intensity from 16 z-stack images of spheroids formed from MiaPaca pancreatic cancer cells (scale bar = 500 µm); (**b**) the quantification of the *Sytox* mean fluorescence intensity +SEM inside the spheroids shaped from initially 3 x 10^3 cells during a 72 h time-course as well as representative images of spheroids from MiaPaca cells during a 72 h time course exposed to the substances B9, (**c**) D7, (**d**) G4 and (**e**) H8 (+/- H_2O_2); (**f**) representative maximum projection intensity images from 16 z-stacks in brightfield channel of Panc01 pancreatic cancer cell spheroids (scale bar = 500 µm); (**g**) quantification of the brightfield channel mean intensity inside the spheroid region at t = 72 h; (**h**) quantification of the destruction area +SEM of loose spheroid formations after incubating the Panc01 spheroids with

B9, (**i**) D7, (**j**) G4 and (**k**) H8 (+/- H_2O_2) with the calculated "area under the curve (AUC)" values to describe the overall differences between the treatment regimen during the whole time-course presented as bar graph. Significance levels for the comparison of substances without H_2O_2 to the respective substances with H_2O_2 (δ), and of their combination (with H_2O_2) to the H_2O_2-alone control (*) were determined via ANOVA. Data are representatives out of five (f-k) or three (a-e) independent replicates.

© 2020 by the authors. Licensee MDPI, Basel, Switzerland. This article is an open access article distributed under the terms and conditions of the Creative Commons Attribution (CC BY) license (http://creativecommons.org/licenses/by/4.0/).
